# Treatment of malignant pericardial effusion with ^32^P-colloid

**DOI:** 10.1038/sj.bjc.6690625

**Published:** 1999-08

**Authors:** W Dempke, N Firusian

**Affiliations:** Department of Internal Medicine IV, Martin-Luther-University Halle-Wittenberg, Ernst-Grube-Str. 40, Halle, 06120, Germany; Department of Medical Oncology and Haematology, Elisabeth Hospital, Roentgenstr. 10, Recklinghausen, 45661, Germany

**Keywords:** pericardial effusion, ^32^P-colloid, response rates

## Abstract

Malignant pericardial effusion is usually treated only when signs of cardiac tamponade develop. Several methods of treatment have been reported with an overall response rate of approximately 75%. Since our initial study using intrapericardial ^32^P-colloid instillation as a treatment modality for pericardial effusion demonstrated a significant higher response rate, this study was conducted to further evaluate the efficacy of intrapericardial ^32^P-colloid in terms of response rates and duration of remissions. Intrapericardial instillation of 185–370 MBq (5–10 mCi) ^32^P-colloid in 36 patients with malignant pericardial effusion resulted in a complete remission rate of 94.5% (34 patients) whereas two patients did not respond to treatment due to a foudroyant formation of pericardial fluid. The median duration time was 8 months. No side-effects were observed. These results suggest that intrapericardial instillation of ^32^P-colloid is a simple, reliable and safe treatment strategy for patients with malignant pericardial effusions. Therefore, since further evidence is provided that ^32^P-colloid is significantly more effective than external radiation or non-radioactive sclerosing agents, this treatment modality should be considered for the management of malignant pericardial effusion. © 1999 Cancer Research Campaign


*British Journal of Cancer* (1999) **80**(12), 1955–1957
© 1999 Cancer Research Campaign
Article no. bjoc.1999.0625

### Treatment of malignant pericardial effusion with $^{32}$P-colloid

**W Dempke[1,2] and N Firusian[2]**

[1]Martin-Luther-University Halle-Wittenberg, Department of Internal Medicine IV, Ernst-Grube-Str. 40, 06120 Halle, Germany; [2]Department of Medical Oncology and Haematology, Elisabeth Hospital, Roentgenstr. 10, 45661 Recklinghausen, Germany

**Summary** Malignant pericardial effusion is usually treated only when signs of cardiac tamponade develop. Several methods of treatment have been reported with an overall response rate of approximately 75%. Since our initial study using intrapericardial $^{32}$P-colloid instillation as a treatment modality for pericardial effusion demonstrated a significant higher response rate, this study was conducted to further evaluate the efficacy of intrapericardial $^{32}$P-colloid in terms of response rates and duration of remissions. Intrapericardial instillation of 185–370 MBq (5–10 mCi) $^{32}$P-colloid in 36 patients with malignant pericardial effusion resulted in a complete remission rate of 94.5% (34 patients) whereas two patients did not respond to treatment due to a foudroyant formation of pericardial fluid. The median duration time was 8 months. No side-effects were observed. These results suggest that intrapericardial instillation of $^{32}$P-colloid is a simple, reliable and safe treatment strategy for patients with malignant pericardial effusions. Therefore, since further evidence is provided that $^{32}$P-colloid is significantly more effective than external radiation or non-radioactive sclerosing agents, this treatment modality should be considered for the management of malignant pericardial effusion.

**Keywords:** pericardial effusion; $^{32}$P-colloid; response rates

Malignant pericardial effusion is known to be a critical complication in cancer patients due to decreased ventricular stroke volume and increased diastolic pressure. Symptoms associated with malignant involvement of the pericardium include dyspnoea, orthopnoea, hepatomegaly, neck vein distension and electrocardiographic changes. The two cancers most frequently associated with malignant pericardial effusion are breast and lung carcinomas, while leukaemia, lymphoma, melanoma and gastrointestinal malignancies are less frequently involved (DeLoach and Haynes, 1953; Skhvatsabaju, 1986). It has been estimated that 35% of patients with lung cancer and 25% of breast cancer patients will be found to have pericardial involvement at autopsy (Shenkai et al, 1982; Buck et al, 1987). The ultimate outcome for patients with malignant pericardial effusion depends on numerous factors, including the performance status, the presence of metastatic disease, the availability of systemic chemotherapy and the local management of pericardial involvement. It is generally accepted that malignant pericardial effusion should only be treated when symptoms of cardiac tamponade develop (Hancok, 1979). During the last two decades several treatment approaches have been reported in the literature, including external radiation therapy (Cham et al, 1975), surgery (pericardial-pleural window and pericardiectomy) (Campbell et al, 1992) or intrapericardial instillation of cytostatic drugs, sclerosing agents or radiocolloids (reviewed by Smith et al, 1974). Although these substances appeared to be superior in managing malignant pericardial effusion, they were still found to be only moderately effective in reducing pericardial fluid formation (Smith et al, 1974). In 1952, Clarke provided the first evidence that malignant pericardial effusion may be controlled by intracavitary application of $^{198}$Au-colloid suggesting that radiocolloids have a significant potential for treatment of pericardial involvement. Recently, two studies using intrapericardial $^{32}$P-colloid for treatment of malignant pericardial effusion have been published (Martini et al, 1977; Firusian, 1980). Both reported high response rates, whereas no complications amongst 39 patients due to the radiocolloid therapy were seen. The encouraging results of these two preliminary studies prompted us to set up a new study using intrapericardial $^{32}$P-colloid instillation to further evaluate the significance of this strategy for treatment of malignant pericardial effusion.

*Received 17 August 1998*
*Revised 14 December 1998*
*Accepted 2 April 1999*

*Correspondence to:* W Dempke

### PATIENTS AND METHODS

$^{32}$P-colloid as a chromic suspension was supplied by Mallinckrodt Nuclear (St Louis, MO, USA). 90% of the particles were in the range of 0.6–2 μm. The physical properties of this compound are shown in Table 1.

Between November 1982 and February 1998 a total of 36 patients with malignant pericardial effusion were selected for intrapericardial $^{32}$P-colloid therapy. Informed consent was obtained from all patients. The study was carried out with ethical committee approval (Muenster University, Germany). All patients developed pericardial effusion while receiving chemotherapy. The patients treated comprised: 23 patients with breast cancer (65%), eight patients with lung cancer (23%) and five patients with other cancers, mainly lymphomas and gastrointestinal tumors (12%). The majority of these patients had received multimodal treatment including chemotherapy and external radiotherapy. In all cases histological and cytological examinations were performed. Median age of the group was 62 years (range 42–86). The

**Table 1** Physical properties of $^{32}$P-colloid

| | |
|---|---|
| Emission | beta |
| Energy (maximal) | 1.71 MeV |
| Energy (mean) | 0.695 Mev |
| Range in tissue (maximal) | 7–8 mm |
| Range in tissue (mean) | 1–4 mm |
| Half-life (physical) | 14.3 days |

**Table 2** Results of intrapericardial $^{32}$P-colloid instillation in 36 patients

| Number of patients | Doses (MBq) | Effect | Duration (months) |
|---|---|---|---|
| 21 | 185 | CR | 8.8 (3–24) |
| 13 | 2 × 185 | CR | 9.3 (3–19) |
| 2 | 2 × 185 | PD | – |

CR: complete remission; PD: progressive disease.

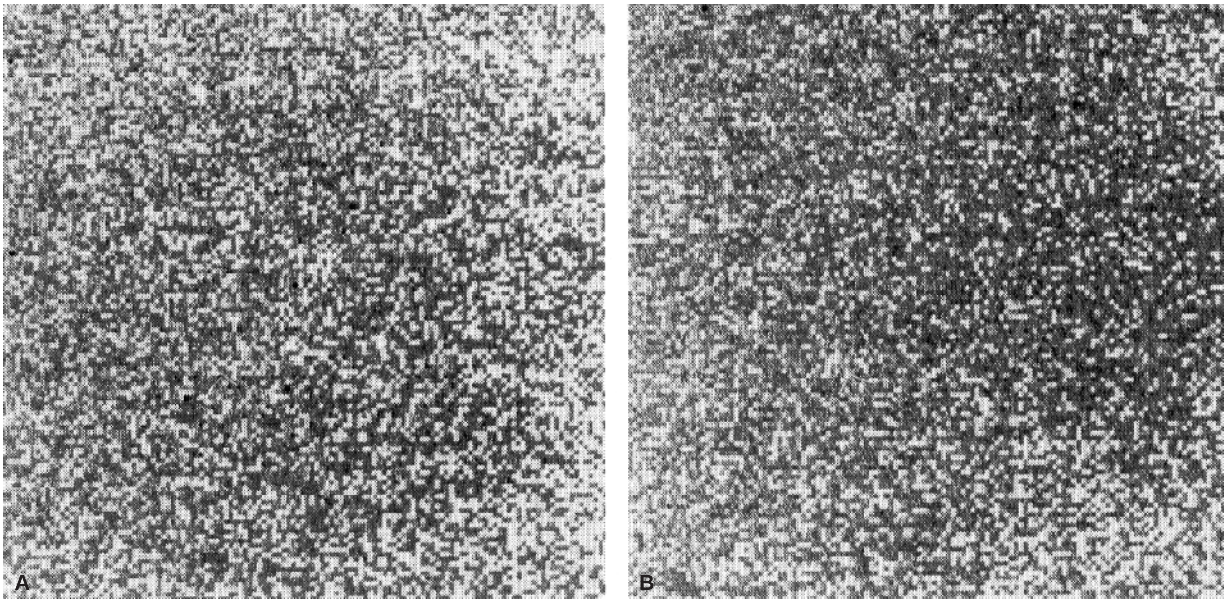

**Figure 1** Typical bremsstrahlung scan following instillation of 5 mCi $^{32}$P-colloid in a patient with breast cancer. (**A**) scan immediately after $^{32}$P-colloid application; (**B**) scan after 4 days following treatment technique for intrapericardial instillation of $^{32}$P-colloid has already been described (Firusian, 1980). Briefly, the appropriate puncture side, angle and depth of insertion was accurately determined by ultrasonography. In addition, the electrocardiography from the needle tip was monitored continously. Following pericardiocentesis, the intrapericardial catheter was left in place, taped securely to the entry side, while radiographic and cytological studies were performed. Prior to $^{32}$P-colloid instillation, care was taken that the pericardial effusion was removed completely. The application of $^{32}$P-colloid was then carried out using the same intrapericardial catheter. According to our initial study (Firusian, 1980) a single dose of 185 MBq (5 mCi) was administered to 21 patients. In 14 patients, however, two injections of 185 MBq within 2 weeks were necessary due to rapid pericardial fluid formation. The catheter was removed immediately after instillation. The distribution of the radiochemical administered was monitored by scintigraphic scans of bremsstrahlung.

In all patients treated, blood samples were analysed for $^{32}$P contamination immediately after drug injection and then twice weekly using a Beckman liquid scintillation counter (Beckman, Munich, Germany).

### RESULTS

Characteristics of patients are listed in Table 2 showing doses instillated and duration of remission. Amongst 36 patients treated, 34 patients achieved a complete, long-lasting remission by $^{32}$P-colloid application (response rate: 94.4%). The median duration of remission was 8 months (range 3–24 months). Two patients with lung cancer, however, did not respond to the pericardial $^{32}$P-colloid instillation (cases 4 and 28); this was due to a rapid formation of pericardial fluid. Both patients died shortly after $^{32}$P-colloid instillation. All patients with a complete remission after intrapericardial $^{32}$P-colloid instillation died due to massive cancer progression; however, in all patients no signs of pericardial effusion were found at time of death as examined by ultrasonography.

In all treated patients serial scintigraphy of bremsstrahlung was performed. The scintigraphic scans demonstrated that a homogenous distribution of the radioisotope had occurred after application (Figure 1). No early complications of intrapericardial radiocolloid therapy were found. In addition, no symptoms of infection or myocardial irritability were observed. In only one patient (case 33)

*© 1999 Cancer Research Campaign*

a transient tachycardia occurred immediately after instillation; however, a cardiac treatment was not necessary. No other side-effects or toxicities were observed.

In all blood samples tested only traces of P-32 activity were found (< 0.3 nCi ml$^{-1}$) indicating that no significant capillary leakage or tissue diffusion had occurred.

### DISCUSSION

Management of malignant pericardial effusion still remains a challenge for every medical oncologist. In the study presented here we have demonstrated that intrapericardial instillation of $^{32}$P-colloid is a highly effective treatment modality in the management of malignant pericardial effusion. Amongst 36 patients treated, the rate of long-lasting complete remissions was 94.5%, confirming the results of the initial study by Firusian (1980).

Since the optimal therapeutic strategy for management of malignant pericardial effusion is still not yet established, several treatment modalities have been reported in the literature. External beam radiation therapy was shown to be effective in treating malignant pericardial effusions in some patients, mainly those with radiosensitive tumours, such as lymphomas and leukaemias which generally respond favourably to radiotherapy (Terry and Klegerman, 1970). However, patients with malignant pericardial effusion secondary to lung or breast cancer responded less favourably. In 38 patients treated with radiotherapy, Cham et al (1975) reported an overall response rate of 61%; the mean duration of relief, however, was only 4 months.

It is generally accepted that surgical intervention should be considered in those patients who did not respond to radiotherapy or intrapericardial therapy. Although the response rates are nearly 100%, a significantly high proportion of patients (13%) developed local recurrence (Mills et al, 1989; Campbell et al, 1992). The mean survival time was approximately 7 months.

Instillations of cytostatic drugs (e.g. bleomycin, 5-fluorouracil, cisplatin) or antibiotics (tetracyclines) have also been described (reviewed in Harbert, 1997). Of these agents, bleomycin and tetracyclines are probably favoured since for both compounds response rates of 70–80% have been reported (Shepherd et al, 1985; van der Gaast et al, 1989). The median duration of remission, however, was only 4 months.

Most recently, Colleoni et al (1998) reported response rates of 23 patients with pericardial effusions after intracavitary treatment with 15 mg thiotepa (days 1, 3 and 5). Nineteen patients (83%) responded to treatment. From this study it was concluded that intracavitary treatment with thiotepa is highly effective and well tolerated in the treatment of malignant pericardial effusion.

However, when compared with other treatment strategies in the management of malignant pericardial effusion, the instillation of $^{32}$P-colloid was found to be superior. Nowrousian et al (1988) treated 16 patients with malignant pericardial effusion by intrapericardial instillation of $^{32}$P-colloid. They found a response rate of 97%, confirming the results we have reported here. Using the method detailed by Harbert (1997) the total dose of beta irradiation following pericardial $^{32}$P-colloid instillation can be estimated. Considering the physical properties of $^{32}$P-colloid (Table 1) and a given distribution volume of the isotope of 1000 ml, a single dose of 5 mCi $^{32}$P would result in a total irradiation dose of more than 100 Gy (Harbert, 1997). Since in standard external beam treatment protocol for radiotherapy of malignant pericardial effusions only up to 30 Gy were delivered (Cham et al, 1977), this dose estimation clearly indicates that intrapericardial $^{32}$P-colloid instillation is more potent in the treatment of malignant pericardial effusions than external beam radiotherapy.

In summary, the results of our study suggest that treatment of malignant pericardial effusion with $^{32}$P-colloid instillation appears to be a simple, highly effective and safe treatment modality. There is evidence that this treatment strategy may be more effective than external radiation therapy or non-radioactive sclerosing agents. Thus, its significant potential should be considered for the management of malignant pericardial effusion.

### REFERENCES

Buck M, Ingle JN and Guilani ER (1987) Pericardial effusion in women with breast cancer. *Cancer* **60**: 263–270

Campbell PT, Van Tright P and Wall TC (1992) Subxiphoid pericardiotomy in the diagnosis and management of large pericardial effusions associated with malignancy. *Chest* **101**: 938–945

Cham WC, Freiman AH and Carstens PHB (1975) Radiation therapy of cardiac and pericardial metastases. *Ther Radiol* **114**: 701–707

Clarke TH (1952) Radioactive colloidal gold198 in the treatment of neoplastic effusions. Northwest U Med School Q Bull **26**: 98–103

Colleoni M, Martinelli G, Beretta F, Marone C, Gallino A, Fontana M, Graffeo R, Zampino G, De Pas T, Cipolla G, Martinoni C and Goldhirsch A (1998) Intracavitary chemotherapy with thiotepa in malignant pericardial effusions: an active and well-tolerated regimen. *J Clin Oncol* **16**: 2371–2376.

DeLoach JF and Haynes JW (1953) Secondary tumors of heart and peritoneum: review of the subject and report of 137 cases. *Arch Intern Med* **91**: 224–230

Firusian N (1980) $^{32}$P-therapy for malignant pericardial effusion. Onkologie **3**: 12–17

Gaast A van der, Kok TC, Hoogerbrugge N and van der Linden TAW (1989) Intrapericardial instillation of bleomycin in the management of malignant pericardial effusion. *Eur J Cancer Clin Oncol* **25**: 1505–1506

Hancock EW (1979) Management of pericardial disease. *Mod Concepts Cardiovasc Dis* **48**: 1–12

Harbert JC (1997) Radiocolloid therapy of malignant pericardial effusion. In: *Principles of Nuclear Medicine*, Harbert JC (ed), pp. 1077–1082. Lippincott: Philadelphia

Martini N, Freiman AH and Watson RC (1977) Intrapericardial instillation of radioactive chromic phosphate in malignant pericardial effusion. *Am J Roentgenol* **128**: 639–644

Mills SA, Holiday RA and Vinten-Johansen JM (1989) Subxiphoid pericardial window for pericardial effusive disease. *J Cardiovasc Surg* **30**: 768–773

Nowrousian MR, Firusian N and Schmidt CG (1988) Diagnostik und Therapie maligner Perikardergüsse. *Beitr Onkol* **33**: 114–144

Shenkai T, Tomenagu K and Saijo N (1982) The incidence of cardiac metastases in primary lung cancer and the management of malignant pericardial effusion. *Jpn J Clin Oncol* **12**: 23–28

Shepherd FA, Ginsberg JS, Evans WK, Scott JG and Oleksiuk F (1985) Tetracycline sclerosis in the management of malignant pericardial effusion. *J Clin Oncol* **3**: 1678–1682

Skhvatsabaju LV (1986) Secondary malignant lesions of the heart and pericardium in neoplastic disease. *Oncology* **43**: 103–109

Smith FE, Lane M and Hudgins PT (1974) Conservative management of malignant pericardial effusion. *Cancer* **33**: 47–56

Terry LN and Klegerman MM (1970) Pericardial and myocardial involvement by lymphomas and leukemias: the role of radiotherapy. Cancer **25**: 1003–1010

*© 1999 Cancer Research Campaign*

## References

[PDF_00249] Buck M., Ingle J. N., Giuliani E. R., Gordon J. R., Therneau T. M. (1987). Pericardial effusion in women with breast cancer.. Cancer.

[PDF_00256] CLARKE T. H. (1952). Radioactive colloidal gold AU198 in the treatment of neoplastic effusions.. Q Bull Northwest Univ Med Sch.

[PDF_00251] Campbell P. T., Van Trigt P., Wall T. C., Kenney R. T., O'Connor C. M., Sheikh K. H., Kisslo J. A., Baker M. E., Corey G. R. (1992). Subxiphoid pericardiotomy in the diagnosis and management of large pericardial effusions associated with malignancy.. Chest.

[PDF_00254] Cham W. C., Freiman A. H., Carstens P. H., Chu F. C. (1975). Radiation therapy of cardiac and pericardial metastases.. Radiology.

[PDF_00258] Colleoni M., Martinelli G., Beretta F., Marone C., Gallino A., Fontana M., Graffeo R., Zampino G., De Pas T., Cipolla G. (1998). Intracavitary chemotherapy with thiotepa in malignant pericardial effusions: an active and well-tolerated regimen.. J Clin Oncol.

[PDF_00262] DELOACH J. F., HAYNES J. W. (1953). Secondary tumors of heart and pericardium; review of the subject and report of one hundred thirty-seven cases.. AMA Arch Intern Med.

[PDF_00264] Firusian N. (1980). 32P-Therapie des malignen Perikardergusses.. Onkologie.

[PDF_00269] Hancock E. W. (1979). Management of pericardial disease.. Mod Concepts Cardiovasc Dis.

[PDF_00273] Martini N., Freiman A. H., Watson R. C., Hilaris B. S. (1977). Intrapericardial installation of radioactive chromic phosphate in malignant effusion.. AJR Am J Roentgenol.

[PDF_00277] Mills S. A., Julian S., Holliday R. H., Vinten-Johansen J., Case L. D., Hudspeth A. S., Tucker W. Y., Cordell A. R. (1989). Subxiphoid pericardial window for pericardial effusive disease.. J Cardiovasc Surg (Torino).

[PDF_00284] Shepherd F. A., Ginsberg J. S., Evans W. K., Scott J. G., Oleksiuk F. (1985). Tetracycline sclerosis in the management of malignant pericardial effusion.. J Clin Oncol.

[PDF_00287] Skhvatsabaja L. V. (1986). Secondary malignant lesions of the heart and pericardium in neoplastic disease.. Oncology.

[PDF_00289] Smith F. E., Lane M., Hudgins P. T. (1974). Conservative management of malignant pericardial effusion.. Cancer.

[PDF_00291] Terry L. N., Kligerman M. M. (1970). Pericardial and myocardial involvement by lymphomas and leukemias. The role of radiotherapy.. Cancer.

[PDF_00266] van der Gaast A., Kok T. C., van der Linden N. H., Splinter T. A. (1989). Intrapericardial instillation of bleomycin in the management of malignant pericardial effusion.. Eur J Cancer Clin Oncol.

